# Using the shuttlebox experimental design to determine temperature preference for juvenile Westslope Cutthroat Trout (*Oncorhynchus clarkii lewisi*)

**DOI:** 10.1093/conphys/coy018

**Published:** 2018-04-18

**Authors:** Camille J Macnaughton, Colin Kovachik, Colin Charles, Eva C Enders

**Affiliations:** Fisheries and Oceans Canada, Freshwater Institute, Winnipeg, Canada R3T 2N6

**Keywords:** photoperiod, shuttlebox, thermal preference, Westslope Cutthroat Trout

## Abstract

Temperature preference for various fishes has often been used as a proxy of optimal temperature for growth and metabolism due to the ease of obtaining preferred temperature zones in laboratory experiments. Several laboratory designs and methods have been proposed to examine preferred temperature zones, however, differences between them (i.e. thermal gradients vs. static temperatures in chambers and duration of acclimation/experimental periods) have led to varying measurements, precluding comparisons between experiments, species and/or life-stages. Juvenile Westslope Cutthroat Trout (*Oncorhynchus clarkii lewisi*), a species listed as threatened in Alberta and of special concern in British Columbia, were tested in an automated shuttlebox experimental design (Loligo® Systems) to determine average and ranges of temperature preference (*T*_pref_) and occupied temperatures. Previous lab studies suggested that Westslope Cutthroat Trout (WCT) prefer temperatures around 15°C, however, we found that average daytime *T*_pref_ for lab-reared juvenile WCT was substantially higher at 18.6°C, with occupied temperatures ranging between 11.9°C and 26.0°C throughout the duration of trials. This seems to indicate that despite constant lab-rearing conditions of 12°C, juvenile WCT may tolerate and even prefer warmer water temperatures. The duration of the acclimation period (1h, 12 h and 24 h) did not have an effect on *T*_pref_, however, *T*_pref_ differed significantly for variable trial durations (12 h, 24 h and 36 h). A closer look at thermal trends throughout trials revealed that photoperiod significantly influenced *T*_pref_, as nighttime temperature preference reached consistently 26°C. Collectively, these results suggest that shuttlebox experiments on WCT need to take into account the photoperiod, as behaviour may drive *T*_pref_ more so than the duration of acclimation periods.

## Teaser

To determine the temperature preference for juvenile Westslope Cutthroat Trout, we conducted an experiment using an automated shuttlebox design (Loligo® Systems), whereby fish regulated ambient temperature by freely moving between cooling and warming chambers. The duration of the acclimation and experimental periods and effect of photoperiod were also evaluated, guiding future thermal preference experiments.

## Introduction

Temperature is a key component shaping fish habitat by setting the limits of thermal niches that provide optimal conditions for growth and other fitness-related activities (Fry, 1947; Magnuson *et al.*, 1979; Jobling, 1981; Chaput *et al.*, 2016). Temperature may also influence fish habitat usage through physiological constraints and temperature preference, driving fish to actively seek specific thermal zones (Breau *et al.*, 2007; DFO, 2012; Petty *et al.*, 2012). Without this mechanism for behavioural homoeostasis, small to large temperature changes can have significant impacts on fish metabolism, among many physiological processes (Breau *et al.*, 2011). Temperature is, therefore, a key component and resource; along with water velocity, depth, substrate and food availability, in determining species-specific suitability of habitats and predicting potential impacts related to habitat changes (Jobling, 1981; Coutant, 1987; Caissie, 2006; Pörtner and Peck, 2010). Consequently, species-specific temperature preference (*T*_pref_) and temperature maxima have been useful proxies for quantifying the optimal temperature zone for growth and metabolism as well as informing temperature limits. *T*_pref_ has been shown to vary across species, size and age, as species and life-stages may require specific temperatures to accomplish physiological functions (i.e. growth) (Fry, 1947; Kwain and McCauley, 1978; Jobling, 1981; Coutant, 1987; Chaput *et al.*, 2016).

Various experimental designs have been developed to determine *T*_pref_ across fish taxa (McCauley *et al.*, 1977; Schurmann *et al.*, 1991; Petersen, 2003; Bear *et al.*, 2007). In these experimental designs, fish are thought to maintain species-specific *T*_pref_ after a period of acclimation (final preferendum; Fry, 1947). However, post-acclimation, fish continue to move and explore temperatures that are above and below *T*_pref_, which is why *T*_pref_ has since been expressed as a temperature zone rather than as a fixed temperature (Jobling, 1981). While there is some evidence that significant differences were not found in *T*_pref_ over a range of acclimation temperatures (McCauley *et al.*, 1977), the length of time fish are allowed to habituate to the shuttlebox or duration of the experimental period has yet to be investigated for the purpose of obtaining reliable *T*_pref_.

In most of the experimental setups used to estimate *T*_pref_, water temperatures are fixed in different quadrants or chambers of the system, allowing the fish to actively seek its *T*_pref_. The presence of confounding variables such as heterogeneous light intensity, availability of perceived cover or changes in water depth in these designs may have also influenced fish behaviour and consequently affected the estimated *T*_pref_ (McCauley *et al.*, 1977; Kwain and McCauley, 1978; Myrick *et al.*, 2004). The annular chamber design by Myrick *et al.* (2004) resolved certain design limitations, most notably by establishing a thermal gradient within the chamber. However, the frequency and duration at which the location of the fish and associated temperature were recorded (every 10 min for an hour), seem relatively coarse and short to establish temperature preference.

Contrary to previous experimental setups with fixed temperature settings, an automated shuttlebox experimental design developed by Loligo® Systems allows for the fish to shuttle back and forth between chambers, thereby adjusting its ambient temperature. This means that fish are not tested within set temperatures; rather, they are allowed to gradually regulate the temperature in the experimental setup. As such, this shuttlebox design may offer a unique opportunity to determine fish thermal preferences and maxima, may consequently prove to be a valuable method to measure continuous thermal profiles for active fishes, and serve as a useful tool in conservation physiology. To date, there are no thermal preference experiments for trout species using this design and little guidance exists on recommended duration for acclimation and experimental periods. Setting methodology guidelines for the length of time allotted for acclimation and experimental periods will collectively serve to guide future thermal preference experiments using the shuttlebox design.

Trout species are known cold water species that behaviourally thermoregulate to select preferred habitats (i.e. temperatures). Bull Trout (*Salvelinus confluentus*), for example, have been shown to select temperatures in the wild between 10 and 15°C in the wild (Gutowsky *et al.*, 2017), while field sampling for Westslope Cutthroat Trout (*Oncorhynchus clarkii lewisi*) suggested a temperature preference between 9 and 12°C (Behnke and Zarn, 1976). Meanwhile, lab studies found that juvenile WCT and Rainbow Trout (*Oncorhynchus mykiss*) shared a similar thermal preference of 14.8–14.9°C when tested in a thermal gradient of 11–17°C and that the optimum growth temperature for WCT was similar to that of Rainbow Trout (13.6°C vs. 13.1°C), recorded over a 60 day testing period (Bear *et al.*, 2007). We hypothesise that *T*_pref_ and range of occupied temperatures for WCT will, therefore, be similar to previous studies conducted for the species or correspond to the temperature at which fish are held prior to experimentation (acclimation temperature of 12°C) (Fry, 1947).

## Methods

### Animal husbandry

WCT used for the experiment were the second generation (F2), hatched from eggs and milt collected from adults originating from the Fording River system in British Columbia, Canada. All fish were reared at the fish holding facility of the Freshwater Institute, Fisheries and Oceans Canada in Winnipeg, Manitoba. Fish were held in holding tanks (200 l) with a continuous inflow of ~12°C water and exposed to a gradual 12/12 light–dark cycle (lights gradually turned on at 07:00 h, were at full light at 08:00 h, and stayed on until 20:00 h). Fish were fed a daily maintenance regime of 1% of the body weight prior to experimentation, then *ad libitum* post-experiment, both times with a combination of high protein trout pellets (EWOS #2 crumble, EWOS Canada Ltd, Surrey, BC, Canada and Martin Mills 3 point food, Martin Mills, Elmira, ON, Canada). All procedures were approved by the Fisheries and Oceans Canada’s Animal Care Committee (Animal Use Protocol # FWI-ACC-2017-03). After each trial, the fish was placed in a separate holding tank, where feeding resumed.

### Experimental setup

**Figure 1:**
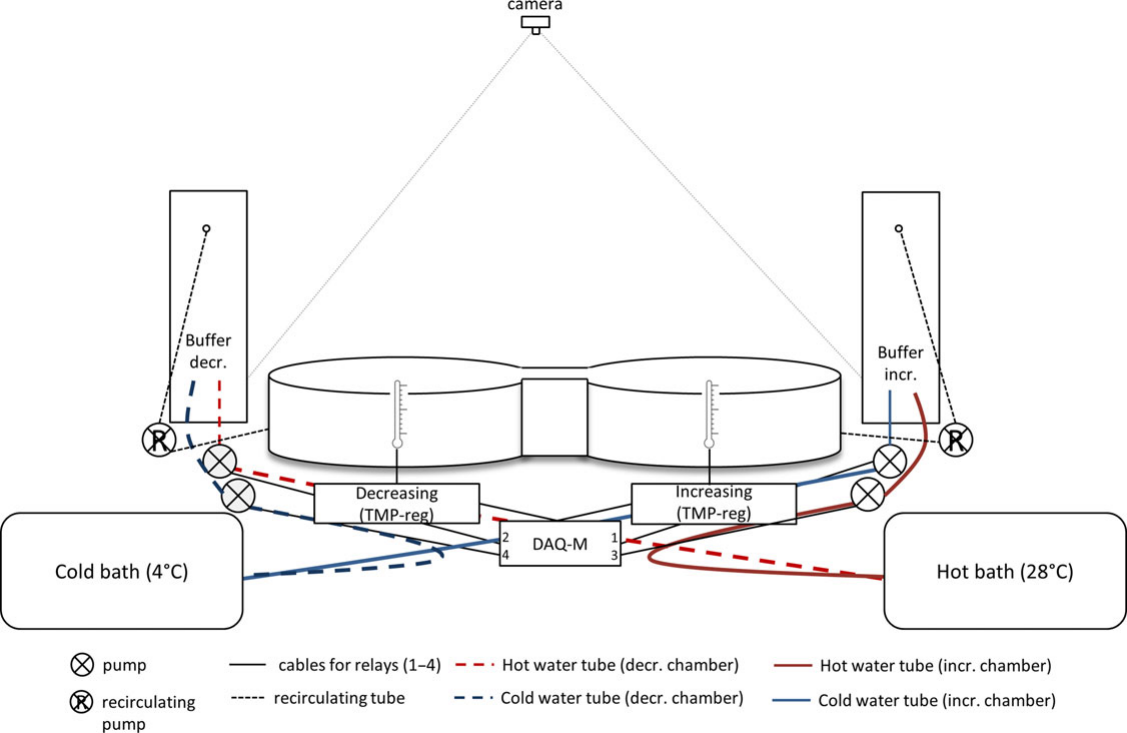
Schematic of the shuttlebox design (chambers and buffer tanks not to scale).

The shuttlebox system consisted of two cylindrical chambers (total system length and width: 110.0 cm × 50.0 cm) connected by a narrow channel (10.0 cm long × 7.5 cm wide) that allows for the unrestricted movement of fish (Loligo® Systems, Tjele, Denmark). Chambers measured 50.0 cm in diameter and water depth in chambers was held around 8.5 cm from the bottom. The overhead camera (uEye 1640-C, Imaging Development Systems, Obersulm, Germany) was anchored to the ceiling ~2 m above the shuttlebox and connected to the motion tracking software (ShuttleSoft behaviour software v.2.6.4, Loligo® Systems). Fish movement was continuously tracked with the overhead camera, relaying the location of the fish in pre-designated chambers, and triggering an increase or decrease in system’s water temperature based on the fish’s position in the increasing or decreasing chamber (Fig. 1). A fish was, therefore, regulating its ambient temperature by shuttling back and forth between chambers until the fish had arrived at a final temperature preference (*T*_pref_). Overhead fluorescent lighting and infra-red light source were used for day and night recording, respectively.

A continuous circular current was maintained in each chamber by pumping water into two raised buffer tanks placed beside the shuttlebox. Water from buffer tanks was allowed to flow gravitationally back into chambers and tanks were insulated using polystyrene foam panels to minimise heat loss or gain with ambient room temperature. Air stones were placed in each buffer tank to increase dissolved oxygen concentration. Temperature probes (Pt-100, accuracy ± 0.15°C) were connected to temperature regulation instruments (TMP-REG, Loligo® Systems) and used to record the temperature within each probe vessel, which were placed in-line between the buffer tanks and shuttlebox chambers. Water temperature was controlled with a series of pumps connected to a DAQ-M instrument that turned on and off through ShuttleSoft, in which temperature limits of 6 and 26°C were set (Loligo Systems 2017). For example, to increase the temperature in a chamber, water was pumped from the buffer tank through a heating coil in a hot water bath held at ~28°C (Heating Thermostat Alpha A 24, Lauda-Brinkmann, Lauda-Königshofen, Germany) and pumped back through the buffer tank to the chamber. Similarly, a cold water bath (Cooling Thermostat Alpha RA 24, Lauda-Brinkmann) was held at ~4°C and served to cool the water in the cooling coil that was connected to the buffer tank and chamber. Water temperature in each chamber was, therefore, independently controlled by the ShuttleSoft, relaying information between the temperature sensors, DAQ-M, and a series of pumps, maintaining desired temperatures between chambers. Due to the leaping ability of WCT, a 1 × 1 cm-fine plastic mesh cover was placed over the shuttlebox to prevent fish escaping. A black-out curtain isolated the shuttlebox system from the rest of the laboratory to minimise disturbance of fish.

### Experimental procedure

Water temperature in each of the shuttlebox chambers did not vary more than 2°C at any given time and were not allowed to change more than 4°C/h. As fish in the holding tanks were held at 12°C, water temperature in chambers was set to 10 and 12°C in the decreasing and increasing chambers, respectively, prior to experimentation. Partial (>50% of the volume) water changes were also done between trials.

Before the start of each trial, fish were measured and weighed. Fifteen juvenile WCT (average total length = 9.41 ± SD 0.79 cm and start wet weight 7.09 ± SD 1.91 g) were tested between 13 June and 2 August 2017. Once water temperature in the chambers stabilised to 10 and 12°C in the decreasing and increasing chambers, respectively, a fish was placed into the shuttlebox, alternating the chamber in which it was placed to exclude any confounding variables associated with the chamber side. The time of day was also recorded at the start and end of the trial to ensure that each trial was initiated during the daytime. All trials except for trial 4 commenced between 9 h 15 min and 13 h 30 min (start time for trial 4: 19 h 50 min).

Once the fish was introduced into the shuttlebox, it was monitored on the ShuttleSoft console to determine if it was exploring both chambers and the camera was accurately tracking its movement. Temperature sensors and pixels/cm were calibrated for each experiment according to the shuttlebox standard operating procedures (Loligo® Systems). Specific to this study, temperature sensors and pixels were calibrated to ±0.15°C and 0.125 pixels/cm, respectively. Preferred temperature (*T*_pref_), water temperature of the chamber occupied by the fish (occupied temperature), and fish swimming speed (cm/s) were recorded every second for each trial. Trials ran for ~48 h. Once the trial was completed, fish wet weight was measured again.

### Data and statistical analyses

Each trial comprised a fish, for a total of 15 trials. Fish weight was compared at the start and end of each trial using Student’s paired *t*-test. Temperature preference has been calculated as the mean selected temperature (Fry, 1947; McMahon *et al.*, 2008), however, preferred temperature (*T*_pref_) was calculated by the ShuttleSoft as the median occupied temperature measured throughout a trial (i.e. cumulative median). Taking the median rather than the average occupied temperature is considered more robust, as the latter metric tends to be biased by extreme or unusual values (Stol *et al.*, 2013). Moreover, a cumulative median in a time series has the benefit of smoothing out short-term fluctuations or in our case, decreasing sensitivity to temperature maxima and provide more stable long-term *T*_pref_ trends. An average *T*_pref_ and standard deviation (SD) was then calculated across trials.

**Figure 2:**
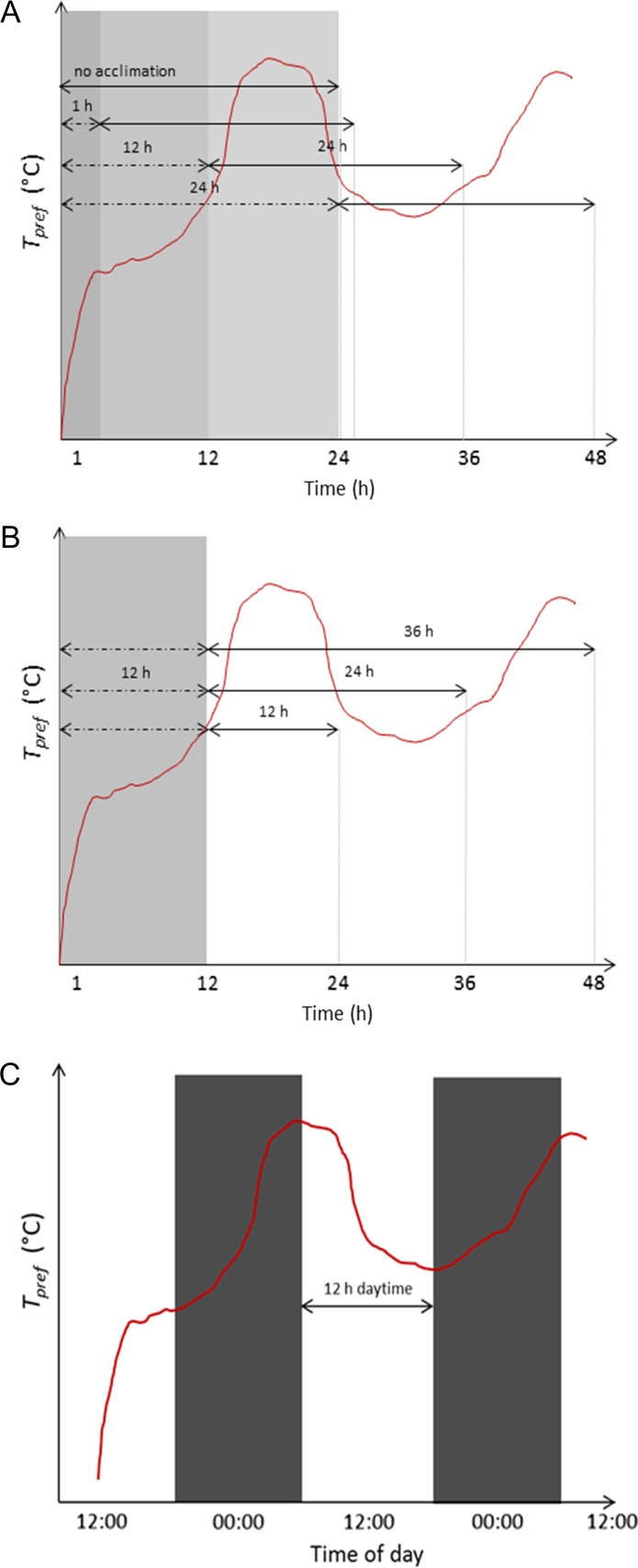
Schematic of *T*_pref_ (cumulative median occupied temperature) over trial duration or time of day to illustrate the different acclimation (**A**), experimental (**B**), and 12 h daytime experimental (**C**) periods evaluated. For a same 24 h experimental period, the first hour, 12 h or 24 h were excluded from calculating *T*_pref_ (A) and for a same 12 h acclimation period, *T*_pref_ was calculated for 12 h, 24 h or 36 h long experiments (**B**).

To test the effect of photoperiod, a non-parametric Randomised Block Design-Friedman rank sum tests, blocking for trial (1–15) was conducted on *T*_pref_ and swimming speed for night (between 20:00 and 08:00 h) and day (between 08:00 and 20:00 h) periods. Assuming that the more a fish is active and shuttling back and forth between chambers, the higher the average swimming speed over the period. Conversely, inactivity or stationary behaviour within a chamber should result in significantly lower swimming speeds. As such, we related swimming speed to fish activity in order to discuss the effect of photoperiod on *T*_pref_. To further evaluate photoperiod differences across trials, *T*_pref_ measured for a 12-h daytime (08:00–20:00 h) was compared with *T*_pref_ values for experiments with equal experimental periods of 12 h (Fig. 2C, Supplemental S2 R code material). About 12-h experimental periods were chosen for comparison due to the 12 h night: 12 h day periods already in place to simulate photoperiod.

Rather than waiting a period of time for fish to adjust to their environment before initiating the software, trials commenced immediately and data recorded over a total of ~48 h. To exclude different acclimation/experimental periods from trials, we recalculated *T*_pref_ as the cumulative median occupied temperature for the desired timeframes since the ShuttleSoft generates *T*_pref_ on all data from the onset of logging. In other words, *T*_pref_ for different acclimation and experimental durations were recalculated by excluding data for specific timeframes (i.e. four different acclimation and three experimental periods each) for each of the 15 trials (Fig. 2A and B). The rationale for selecting these different acclimation and experimental periods stem from previous temperature preference experiments conducted in a laboratory setting (Myrick *et al.*, 2004; Bear *et al.*, 2007). For the same duration of experimental period (24 h), we calculated *T*_pref_ without an acclimation period, excluding the first hour, 12 and 24 h of occupied temperatures recorded per trial (acclimation periods; Fig. 2A). Photoperiod for different acclimation period was consistent across trials (12 h night: 12 h day). For the same acclimation period (0 or 12 h), we calculated *T*_pref_ for experimental periods of 12, 24 and 36 h (Fig. 2B). Since experiments were initiated at different times during the day, photoperiod for different experimental periods varied across trials. Specifically, average daytime among trials was 8 h 30 min ± SE 38 min and 20 h 20 min ± SE 37 min for 12 and 36 h experimental periods without acclimation, respectively. Likewise, average daytime among trials post-acclimation (12 h) was 4 h 44 min ± SE 40 min and 15 h 52 min ± SE 45 min for 12 and 36 h experimental periods, respectively. It follows that for equal experimental periods of 12 h (Fig. 2C), average daytime differed from 12 h daytime. *T*_pref_ calculated for different acclimation and experimental durations were averaged across trials (Table 1).

**Table 1:** Summary of average and range of *T*_pref_ (cumulative median occupied temperature) for different acclimation and experimental periods

Duration of the acclimation period (h)	Duration of the experimental period	Average *T*_pref_ ± SD (°C)	Range of *T*_pref_ (°C)
0	12 h (daytime)	18.6 ± 0.7	17.7–20.6
	12	20.5 ± 1.8	18.0–25.6
	24	22.0 ± 0.8	20.5–23.7
	36	21.1 ± 1.4	19.4–22
	48	20.9 ± 2.2	12.1–24.6
1	12 h	20.5 ± 1.8	17.8–25.6
	24 h	22.0 ± 0.8	20.5–23.7
	36 h	21.1 ± 1.4	19.4–22.0
12	12 h	24.8 ± 1.8	18.8–25.7
	24 h	22.0 ± 0.9	20.7–24.0
	36 h	24.2 ± 1.4	20.9–25.2
24	24 h	22.8 ± 1.9	18.5–25.6

A non-parametric Randomised Block Design-Friedman rank sum test, blocking for trial (1–15) followed by a post hoc Kruskal–Wallis multiple comparison were conducted on *T*_pref_ to test the effect of acclimation/experimental periods overall trials. P-values for the post hoc test were adjusted with the Benjamini–Hochberg method (Dunn, 1964) and the analyses were blocked for trial to account for the dependence among observations. To further evaluate the effect of photoperiod among experimental periods, Randomised Block Design-Friedman rank sum tests were conducted on daytime, a continuous variable (min) quantified per experimental period. All database manipulation and statistical analyses were done in R version 3.4.0 (R Core Team, 2017).

## Results

**Figure 3:**
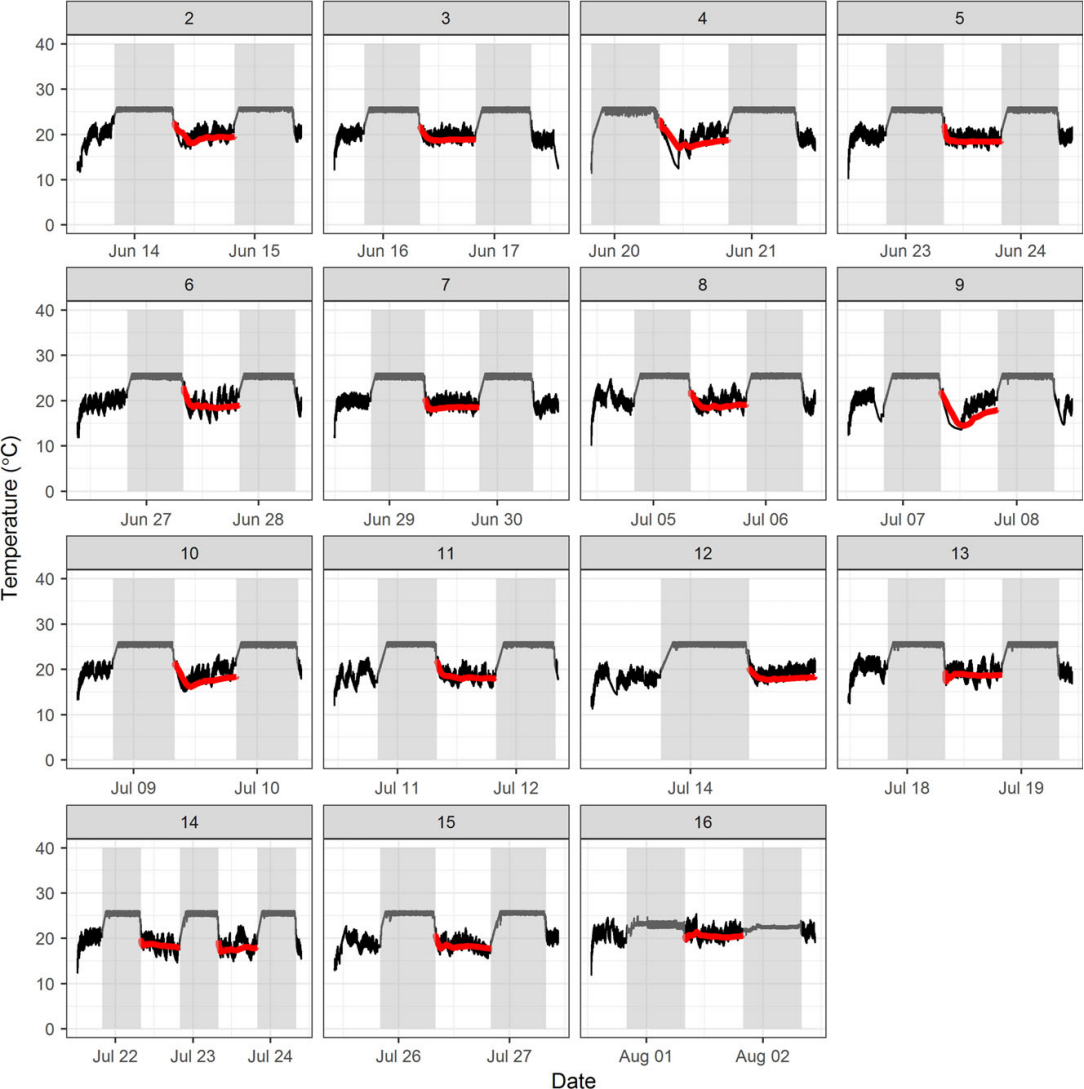
Occupied temperature over time (black line) and *T*_pref_ for 12 h daytime (08:00–20:00 h) (red line) per trial. Grey boxes correspond to night periods (20:00–08:00 h).

Fish weights significantly decreased from start to end of the trials (average difference = 0.20 g), but remained highly correlated (*r* = 0.99). In general, juvenile WCT were seen to occupy temperatures between 11.9 and 26.0°C (average = 21.9 ± SD 3.5°C) when allowed to behaviourally thermoregulate over an experimental temperature gradient of 20°C. For this same time period, average *T*_pref_ overall trials was 20.9 ± SD 2.2°C, however, thermal patterns tracked diel changes, with significantly higher overnight average *T*_pref_ (21.4 ± 0.9°C) than during the day (20.4 ± 1.0°C) (Friedman *χ*^2^ = 15, df = 1, *P* < 0.005). Average swimming speed was also significantly lower at night vs. day (Friedman *χ*^2^ = 11.27, df = 1, *P* < 0.005), with average night swimming speeds of 2.57 ± 3.02 cm/s vs. day swimming speeds of 6.90 ± 3.12 cm/s. Based on thermal profiles, fish did not appear to shuttle back and forth between chambers at night, selecting the increasing chamber to rest and consequently increasing *T*_pref_ (Fig. 3). We inspected the experimental setup and infra-red lighting throughout the experiment to confirm that the camera tracked the fish in the shuttlebox at night. On occasion, however, the luminosity/contrast needed to track the fish at night was not sufficient to accurately track nighttime shuttling behaviour, which may explain this apparent diel fluctuation. Unfortunately, trials are not video recorded with ShuttleSoft for verification. As such, an upper occupied temperature of 26.0°C observed at night suggests that it may result from the experimental design (i.e. upper temperature setting for the system) rather than an indication of the species’ upper thermal tolerance. Consequently, *T*_pref_ measured for the period of time where fish were observed to shuttle between chambers (i.e. 12 h daytime), was deemed more reliable than *T*_pref_ measured throughout the duration of the experiment (18.6 ± 0.7°C; Table 1 and Fig. 3).

**Figure 4:**
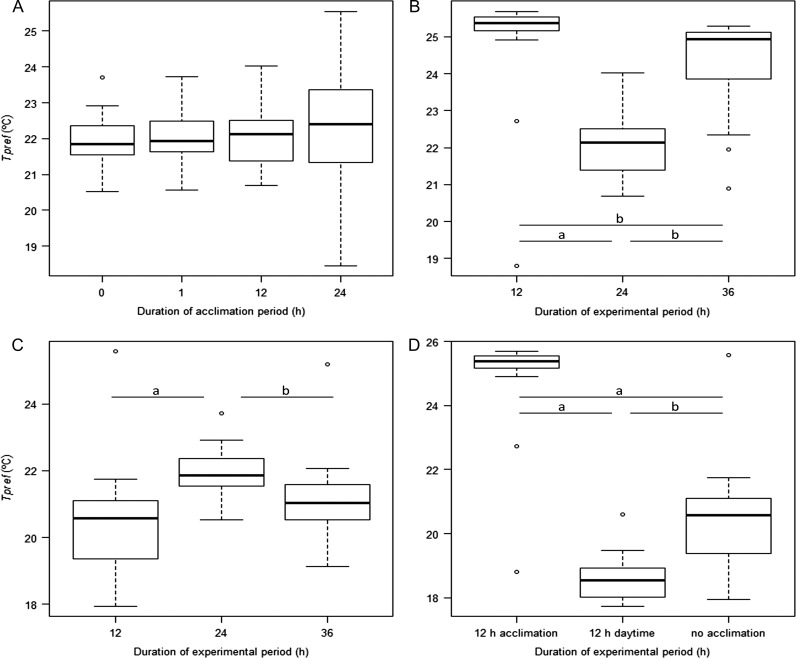
Boxplots of *T*_pref_ (°C) by acclimation and experimental periods: *T*_pref_ for 0, 1, 12 or 24 h of acclimation followed by a 24 h experimental period (**A**); *T*_pref_ for 12 h (**B**) or no (**C**) acclimation followed by 12, 24 or 36 h experimental periods and *T*_pref_ for 12 h experimental periods, including 12 h daytime (**D**). Significant differences for post hoc tests are indicated by *a* (Adj. *P* < 0.005) and *b* (Adj. *P* > 0.01).

*T*_pref_ did not vary significantly between different acclimation periods (Friedman *χ*^2^ = 6.57, df = 3, *P* = 0.08; Fig. 4A), but varied significantly whether the duration of the experiment was 12, 24 or 36 h long. Specifically, for a constant acclimation time of 12 h, *T*_pref_ varied significantly between all three experimental periods (Friedman *χ*^2^ = 20.37, df = 2, *P* < 0.005; Fig. 4B). This difference is partly due to the photoperiod as average daytime varied from 4 h 40 min to 15 h 52 min. In fact, photoperiod (daytime) varied significantly with experimental periods (Friedman *χ*^2^ = 30, df = 2, *P* < 0.005). Likewise, when the acclimation period was excluded altogether when testing different experimental periods (i.e. 0 h acclimation for 12, 24, 36 and 48 h experimental periods), differences among *T*_pref_ were significant (Friedman *χ*^2^ = 22.53, df = 2, *P* < 0.005), but only between experimental periods 12 and 24 h (Adj. *P* < 0.005) and 24 h and 36 h (Adj. *P* = 0.03; Fig. 4C). Average daytime differed 3 h 30 min between 12 and 24 h experimental periods, whereas it varied 9 h between 24 and 36 h experimental periods. In addition, the spread of *T*_pref_ values between experimental periods for 0 and 12 h acclimation periods was substantially decreased (Fig. 4B and C), with none of the values for 0 h acclimation surpassing 22°C vs. 26°C for 12 h acclimation (Table 1). *T*_pref_ for a 12 h daytime (08:00–20:00 h, inclusively) was contrasted with *T*_pref_ measured for similar 12 h experimental periods (i.e. 0 and 12 h acclimation periods followed by a 12 h experimental period; Fig. 4D). *T*_pref_ was significantly different among 12 h timeframes (Friedman *χ*^2^ = 24.7, df = 2, *P* < 0.005), however, the lowest *T*_pref_ across all acclimation and experimental scenarios was measured for the 12 h daytime (18.6 ± 0.7°C; Table 1). Again, significant differences in photoperiod were observed among 12 h timeframes (Friedman *χ*^2^ = 28.13, df= 2, *P* < 0.005).

## Discussion

The current shuttlebox design characterised the thermal biology of WCT by testing lab-reared fish in an environment where lighting, water depth and perceived cover were uniform in the experimental arena and among trials, and therefore eliminating previously identified confounding factors. Fish were unrestricted and regulated temperature by shuttling back and forth freely between chambers, allowing for greater flexibility in fish behaviour than systems with static temperatures in quadrants. As such, we suspect that WCT in our study were not constrained by imposed upper temperature limits (17°C; Bear *et al.*, 2007) that may in fact turn out to be well within the species’ thermal range. Furthermore, data was continually recorded for up to 48 h, depicting real-time thermal and activity profiles for each trial, where *T*_pref_ for different acclimation and experimental periods may be compared to refine the experimental protocol. An advantage of running experiments for 48 h rather than for shorter experimental periods (e.g. 1 h; Myrick *et al.*, 2004) is that we observed diurnal differences in *T*_pref_ that may have otherwise been missed.

The shuttlebox setup and ShuttleSoft were reliable and autonomous, however, the frames per second (FPS) decreased the longer the experiment ran, causing a couple second lag with the display on the computer software nearing the end of the 48 h trials. Lower FPS may have influenced the distance travelled by fish, but overall occupied temperature and swimming speed trends appeared consistent between trials. Moreover, the way that *T*_pref_ was calculated by ShuttleSoft as the cumulative median of occupied temperatures may limit certain data manipulations from output files (e.g. excluding or sub-setting specific timeframes). However, by recalculating *T*_pref_ at the same timescale(s) as the ShuttleSoft, as we have done, we were able to manipulate data after the experiments were completed (Supplemental S2 R code material), rather than risk shutting down the ShuttleSoft recording during the experiment or stressing out fish in the chambers.

The time allotted for fish to habituate to experimental setups in behavioural thermoregulatory studies varies substantially, from no time for Chinook Salmon smolts (*Oncorhynchus tshawytscha*) in experiments conducted by Sauter *et al.* (2001), overnight for juvenile Rainbow Trout (McCauley *et al.*, 1977), to 12 h for less active fish species like Lake Sturgeon (*Acipenser fulvescens*) or 24 h for a relatively active fish species like Carmine Shiner (*Notropis percobromus*) (Stol *et al.*, 2013). Likewise, to ensure that sufficient data is recorded for reliable measurements, the general consensus is longer experiments are better, but experimental guidelines remain vague. As it stands, the duration of both periods has not been determined for trout species in the current shuttlebox design. In the present study, we showed that for a same experimental period, *T*_pref_ did not significantly differ across acclimation periods, suggesting that habituation to the experimental setup was fairly quick and fish actively controlled temperature within a short time period. However, whether the experiment ran for 12, 24 or 36 h, *T*_pref_ varied significantly among experimental periods, indicating that fish occupied different temperatures throughout the experiment.

There is evidence that photoperiod, age and size may affect temperature preference of salmonid species (Kwain and McCauley, 1978; Coutant, 1987; Sauter *et al.*, 2001). Indeed, our results showed significant diel fluctuations in activity behaviour, with increased average *T*_pref_ and reduced swimming speed from 20:00 to 08:00h, suggesting that juvenile WTC may have selected the warmer chamber to rest in overnight. While fish tracking was inspected for night and day periods prior to each trial, the fact that *T*_pref_ consistently increased up to a maximum of 26.0°C across trials suggests there may have been some delays attributed to lower contrast between the fish and its surroundings. As such, WCT were potentially constrained by imposed upper temperature limits. Variations in start time across trials led to differences in photoperiods for experimental periods 12 and 36 h, which explain, in part, significant *T*_pref_. Overall, the more daytime included in the timeframe used to calculate experimental periods, the lower the median *T*_pref_ and spread of values across trials. If only daytime occupied temperatures are considered, *T*_pref_ was lower (18.6 ± 0.7°C) than any other acclimation and/or experimental treatment, and closer to *T*_pref_ values observed in other laboratory experiments (Bear *et al.*, 2007). These results collectively point to the importance of photoperiod when running thermal biology experiments on trout species in the current shuttlebox design. Specifically, we propose that experiments for juvenile WCT run for equal total night and day duration, paying attention to nighttime shuttling behaviour, or that *T*_pref_ be calculated for daytime periods only to represent active seeking periods. In our study, the latter option was deemed more reliable and *T*_pref_ for lab-reared juvenile WCT was determined at 18.6°C. In addition to photoperiod, sensitivity to elevated temperatures is potentially higher in larger individuals than smaller individuals (Rodnick *et al.*, 2004; Pörtner and Knust, 2007; Rijnsdorp *et al.*, 2009), supporting the notion that juvenile or smaller WCT may be less sensitive to higher temperatures or greater temperature ranges. Given that the lab-reared juvenile WCT used in this study had not experienced natural diel or seasonal fluctuations in temperature and photoperiod, it might be said that *T*_pref_ measured may not truly reflect *T*_pref_ for wild fish. Therefore, a comparison of thermal biology among juvenile and adult WCT, in addition to lab-reared vs. wild fish, is needed before drawing conservation guidelines for the species as it pertains to describing temperature limits for their habitat.

Understanding the thermal sensitivity of trout species, especially listed threatened or special concern species under the Canadian *Species at Risk Act*, is of the utmost importance, as increasing water temperatures and altered seasonal thermal regimes in their habitat grows. Preferred temperatures are well correlated with optimal temperature for growth and metabolism and as such, are often used as proxies in bioenergetics modelling (Jobling, 1981). Contrary to much of the literature, juvenile WCT in our study appeared to occupy higher temperatures throughout the experiment, preferring on average a range of temperatures between 17.7 and 20.6°C. In fact, this temperature preference zone falls within the upper temperature tolerance range previously observed for the species (19.1–20.3°C; Bear *et al.*, 2007). Despite the fact that *T*_pref_ was greater than expected, the range of average *T*_pref_ was narrow at Δ*T*_pref_ = 2.9°C, supporting the notion that the *T*_pref_ zone should narrow over time (Fry, 1947; Stol *et al.*, 2013). However, Δ*T*_pref_ or preferred temperature zone largely depended on how long *T*_pref_ was measured.

In the field, preferred temperatures for WCT are thought to range from 9 to 12°C (Behnke and Zarn, 1976) and the species’ preference for cooler water temperatures has been shown to drive their occupancy in cooler headwater streams (i.e. higher elevation), where physiological demands are decreased (Fausch, 1989; Paul and Post, 2001; Rasmussen *et al.*, 2010). In Canada, WCT has historically occupied a wide range of habitats, from headwaters downstream to the plains, however, it now exhibits a fragmented distribution over most of its range (Cleator *et al.*, 2009). Consequently, if *T*_pref_ of 18.6°C should be within the optimal temperature range for juvenile WCT growth, it would suggest that WCT could inhabit much warmer waters than initially thought, as they did in the past. As water temperature in streams and rivers fluctutates seasonally and diurnally, we would expect that trout species adapted to these temporally variable environments would exhibit wide range of temperature preference, without incurring physiological impacts on individual fitness. Current distribution of WCT in colder headwaters may, therefore, likely be driven by predation at early life-stages, inter-specific competition with invasive trout species (Rainbow Trout, Brook Trout (*Salvelinus fontinalis*), and Brown Trout (*Salmo trutta*)), and stocking of WCT, rather than the species preference for cold water habitats.

The automated shuttlebox system demonstrated a greater ability to account for fish thermoregulatory behaviour, while continuously tracking temperature preference and activity of fish over time. In addition, the shuttlebox system can also be used to analyse the effect of a range of other environmental factors including water turbidity, salinity and oxygen saturation, further extending the system’s usefulness as a tool in conservation physiology. Assuming that fish are adapted to the thermal regime of their environment, their temperature preference should fall within the range of their natural habitat (Pörtner and Farrell, 2008). This understanding can be applied to develop a mechanistic comprehension of how fish populations may react to environmental changes and can be particularly useful for bioenergetics habitat modelling (Rosenfeld, 2003), niche modelling (Kearney and Porter, 2009), and individual based modelling (Railsback *et al.*, 2005), in situations of advising conservation action and management planning.

## Supplementary Material

Supplementary Data
