# Supplementary material for: Using the shuttlebox experimental design to determine temperature preference for juvenile Westslope Cutthroat Trout (Oncorhynchus clarkii lewisi)
Source: Conserv Physiol. 2018 Apr 18;6(1):coy018. doi: 10.1093/conphys/coy018 (PMC5906927; doi:10.1093/conphys/coy018)
Supplement: Supplementary Data [file coy018supplementalmaterial.cons.phys.revised.docx]

**Supplemental material:**

**S1.** Mean occupied temperature by hour (black line), mean increasing and decreasing chamber temperatures by hour (grey range), median occupied temperature by hour (blue line), and *T_pref_* (red line) for trial 3.


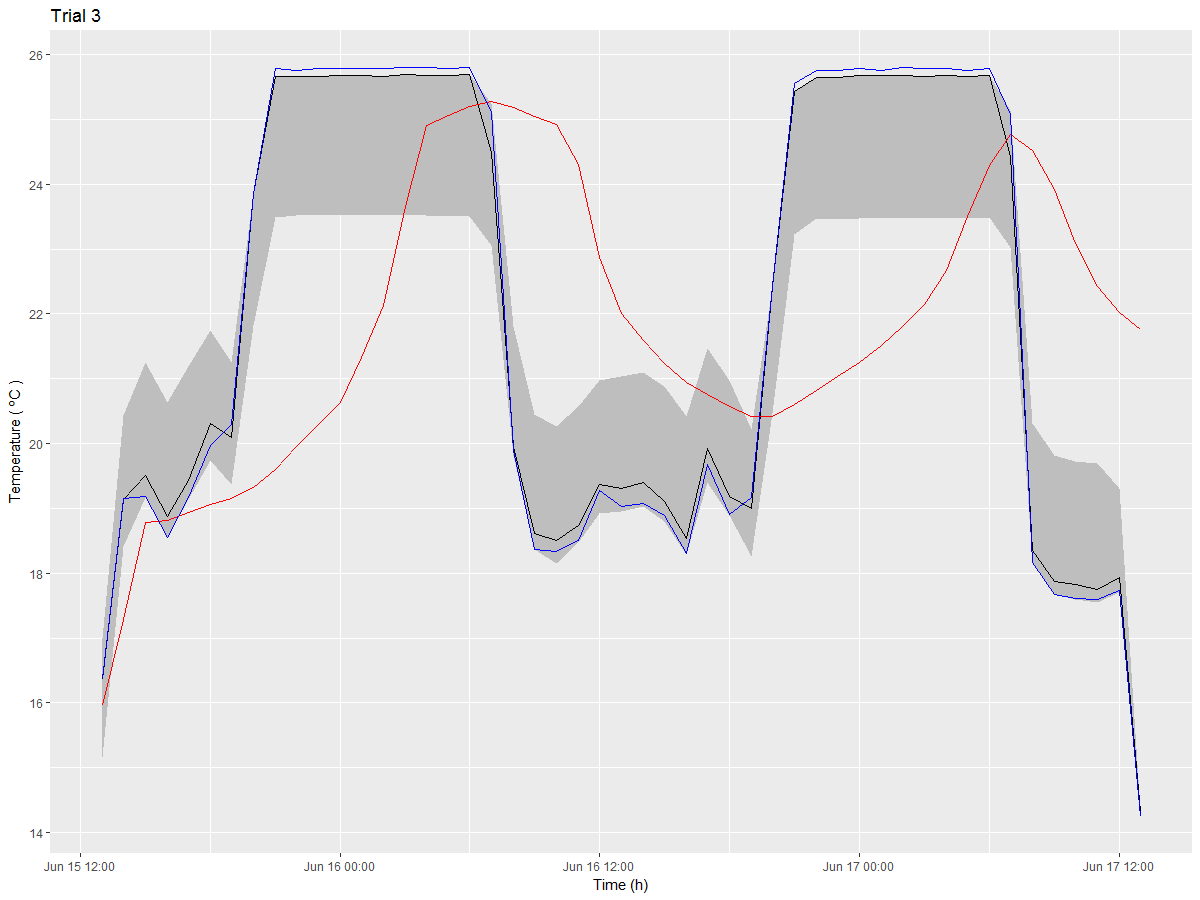


**S2.** R-code to calculate preferred temperature *T_pref_*.

### Code Section

#####

# Run daytime period and calculate the running medians

data1 = data %>%

  group_by(trial) %>%

  select("trial","dateTime","obj.temp","pref.temp") %>%

  mutate(Day = format(dateTime, format = "%d-%b-%Y")) %>%

  filter(Day == unique(Day)[2]) %>%

  mutate(hour = as.numeric(format(dateTime, "%H"))) %>%

  filter(hour > 7) %>%

  do(accPeriod(., acclimation = 0, exposure = 12))

data2 = data %>%

  filter(trial == 14) %>%

  select("trial","dateTime","obj.temp","pref.temp") %>%

  mutate(Day = format(dateTime, format = "%d-%b-%Y")) %>%

  filter(Day == unique(Day)[3]) %>%

  mutate(hour = as.numeric(format(dateTime, "%H"))) %>%

  filter(hour > 7) %>%

  do(accPeriod(., acclimation = 0, exposure = 12))

test = rbind(as.data.frame(data1), data2)

night = data.frame(start = seq.POSIXt(from = as.POSIXct("2017-06-01 20:00:00"),

                                      to = as.POSIXct("2017-08-31 20:00:00"),

                                      by = "day"),

                   end = seq.POSIXt(from = as.POSIXct("2017-06-02 8:00:00"),

                                      to = as.POSIXct("2017-09-01 8:00:00"),

                                      by = "day"),

                   dateTime = seq.POSIXt(from = as.POSIXct("2017-06-01 20:00:00"),

                                         to = as.POSIXct("2017-08-31 20:00:00"),

                                         by = "day"),

                   obj.temp = 1,

                   trial = NA)

nightTime = NULL

for(i in 1:nrow(night)){

  df = night[i,]

  for(j in unique(data$trial)){

    df2 = data[data$trial == j,]

    if(df$start %in% df2$dateTime | df$end %in% df2$dateTime){

      df$trial = j

      nightTime = rbind(nightTime,df)

    }

  }

}

rm(df, df2, i ,j)

data %>%

  left_join(.,test[,c("dateTime","run.med")]) %>%

  # mutate(night = ifelse(hour < 8 & hour >= 20, 0, 1)) %>%

  ggplot(., aes(dateTime, obj.temp)) +

  geom_path() +

  geom_path(aes(y = run.med),col = 2, lwd = 1.5) +

  facet_wrap(~trial, scales = "free_x") +

  scale_x_datetime(breaks = seq.POSIXt(from = as.POSIXct("2017-06-01"),

                                       to = as.POSIXct("2017-08-31"),

                                       by = "day"),

                   labels = date_format("%b %d")) +

  xlab("Date") + ylab(expression("Temperature (" * degree * "C)")) + theme_bw() +

  geom_rect(data = nightTime, aes(xmin = start, xmax = end, ymin = 0, ymax = 40),

            fill = "grey", alpha = 0.5)

ggsave("Daytime Running Medians.tiff", dpi = 400, height = 20, width = 20,

       units = "cm", compression = "lzw")

# write the csv

test %>%

  group_by(trial,Day) %>%

  dplyr::summarise(final_value = last(run.med)) %>%

  write.csv(.,file = "Final Daytime Cumulative Medians.csv", row.names = F)
